# A retrospective analysis of 38,652 amniotic fluid karyotype

**DOI:** 10.3389/fgene.2025.1655290

**Published:** 2025-08-15

**Authors:** Jianyu Ren, Xiaojiao Guan, Wenzhe Lv, Yousheng Yan, Yanmei Si, Shufa Yang, Chenghong Yin

**Affiliations:** Prenatal Diagnostic Center, Beijing Obstetrics and Gynecology Hospital, Capital Medical University, Beijing Maternal and Child Healthcare Hospital, Beijing, China

**Keywords:** prenatal diagnosis, amniocentesis, karyotype analysis, chromosomal aberrations, database

## Abstract

**Background:**

Chromosomal karyotype analysis remains a classical and frontline method in prenatal diagnosis, capable of detecting balanced chromosomal abnormalities and providing insights distinct from high‐resolution molecular techniques such as CMA and CNV‐Seq. However, large‐scale studies on the distribution of structural abnormalities and mosaicism in amniotic fluid karyotypes are scarce, with most previous research focusing on common aneuploidies.

**Objective:**

The study aimed to elucidate the relationship between chromosomal structural abnormalities and specific chromosomes.

**Methods:**

We established a large‐scale amniotic fluid karyotype database by collecting prenatal diagnostic indications and karyotype analysis results from amniotic fluid samples of 38,652 pregnant women who underwent prenatal diagnosis at the Beijing Obstetrics and Gynecology Hospital.

**Results:**

From 2010 to 2024, the proportion of high-risk serological screening cases showed a decreasing trend year by year, while the proportions of high-risk non‐invasive prenatal testing, increased nuchal translucency, and ultrasound abnormalities all showed increasing trends. Among all results, the proportions of non‐mosaic abnormalities, mosaicism, polymorphisms, and normal karyotypes were 4.68%, 0.71%, 1.7%, and 92.91%, respectively. Inversion of chromosome 9 and variations in heterochromatin length of the Y chromosome were the most common polymorphisms. Sex chromosome aneuploidies were more prone to mosaicism. Inversions of chromosomes 9 and Y were the most frequent types of inversions. Robertsonian translocations occurred most commonly between chromosomes 13 and 14, while reciprocal translocations were most frequently observed between chromosomes 11 and 22. Chromosome breakage was most common in chromosomes Y and 1, whereas deletions were most frequently detected in chromosomes X and 5. Isochromosomes mainly appeared in a mosaic form in chromosome X. Among all indication groups, high-risk NIPT was associated with the highest positive rate for unbalanced abnormalities. A searchable karyotype database was setup, which allows users to query abnormal karyotypes identified in this study.

**Conclusion:**

Specific chromosomal abnormalities and mosaicisms tend to occur in particular chromosomes. Therefore, attention should be paid to specific chromosomes during karyotype analysis.

## 1 Introduction

Chromosomal karyotype analysis of amniotic fluid cells remains a classical method for diagnosing chromosomal disorders in prenatal diagnosis. High-resolution molecular techniques such as chromosomal microarray analysis (CMA) and copy number variation sequencing (CNV-Seq) have significantly improved the detection rate of genetic etiologies in fetuses with developmental abnormalities (Xia et al., 2020; Wang J. et al., 2022; Zhuang et al., 2024). Compared to CMA and CNV-Seq, karyotype analysis has lower resolution. However, it offers the unique advantage of detecting balanced chromosomal abnormalities and determining the specific location of these abnormalities. As such, karyotype analysis provides insights into genomic alterations from a perspective distinct from that of CMA and CNV-Seq, reflecting population-level patterns of chromosomal abnormalities. At present, karyotype analysis remains a frontline tool in prenatal genetic testing.

Due to the wide variety of chromosomal structural abnormalities, studies with smaller sample sizes often lack the statistical power to observe recurring structural variants. Consequently, previous research on amniotic fluid karyotype analysis has primarily focused on the detection rates of common chromosomal abnormalities across different clinical indications. These commonly reported abnormalities include aneuploidies, deletions, duplications, inversions, derivative chromosomes, and isochromosomes (Wang LF. et al., 2022; Zheng et al., 2024; Nishiyama et al., 2015). Structural abnormalities, unlike aneuploidies, are rarely analyzed at the level of specific chromosomes. Moreover, large-scale studies exploring mosaicism in the amniotic fluid population are lacking, making it difficult to understand the distribution of mosaicism and its correlation with specific chromosomes (Hao et al., 2020).

To address these gaps, we analyzed data from 38,652 patients who underwent chromosomal karyotype analysis of amniotic fluid cells at the Prenatal Diagnosis Center of Beijing Obstetrics and Gynecology Hospital between January 2011 and June 2024. We performed a comprehensive statistical analysis based on prenatal diagnostic indications, mosaicism, chromosomal structural classification, chromosomal involvement, and chromosomal balance. Finally, we established a searchable karyotype database that allows users to query karyotype types by chromosome: https://yangsf.shinyapps.io/karyotype_search/.

## 2 Materials and methods

### 2.1 Subjects

The study included pregnant women who underwent prenatal diagnosis at Beijing Obstetrics and Gynecology Hospital between January 2011 and June 2024. Inclusion criteria were: (1) provision of signed informed consent; and (2) successful cell culture with available chromosomal karyotype analysis results.

### 2.2 Clinical data collection

Clinical information was collected from pregnant women undergoing prenatal diagnosis, including maternal age, date of prenatal diagnosis, indications for prenatal diagnosis and results of amniotic fluid chromosomal karyotype analysis.

### 2.3 Karyotype analysis of amniotic fluid

The karyotype analysis of amniotic fluid primarily involved three steps: cell culture, slide preparation and G-banding, and microscopic analysis. The detailed procedures have been described in previous studies (Yang et al., 2018).

### 2.4 Statistical analysis

**FIGURE 1:**
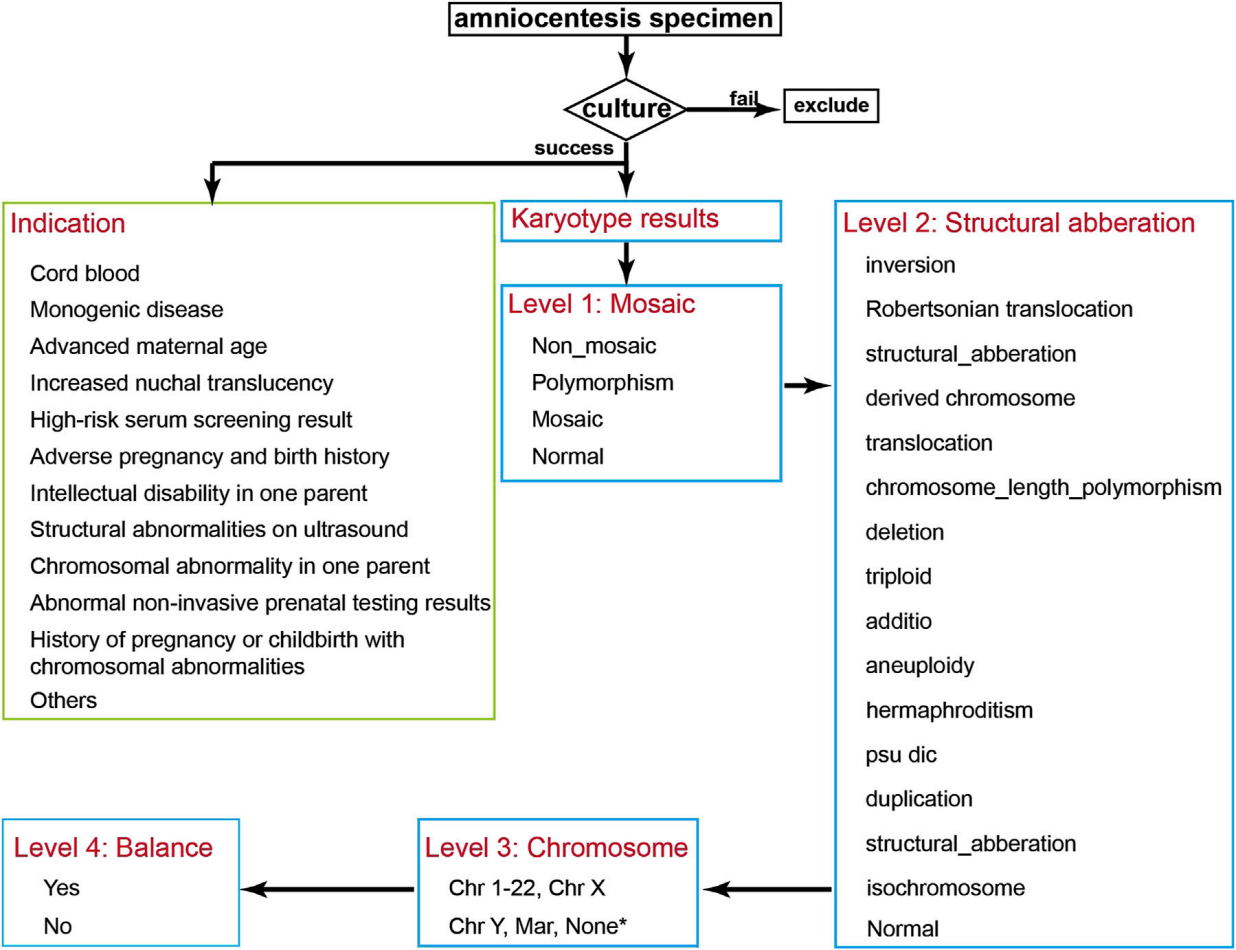
Data Analysis Flowchart. After collecting patient data, samples with failed cultures are excluded, and the prenatal indications for the pregnant women are classified. The chromosomal karyotype results are then classified sequentially into Level 1 to Level 4 categories. * Hermaphroditism, triploid, and normal karyotypes were annotated as “None”.

After collecting the clinical data of pregnant women undergoing prenatal diagnosis, statistical analysis was performed using R software (version 4.4.2). Data visualization was conducted with the ggplot2 package. The analysis focused on prenatal diagnostic indications and karyotype results. The analytical workflow is illustrated in Figure 1.

Prenatal diagnostic indications were categorized into the following groups: advanced maternal age, high-risk serum screening result, increased nuchal translucency (NT), high-risk non-invasive prenatal testing (NIPT), structural abnormalities on ultrasound, chromosomal abnormality in one parent, history of pregnancy or childbirth with chromosomal abnormalities, intellectual disability in one parent, monogenic disease, cord blood, adverse pregnancy and birth history, and others. The definitions of each category are as follows:

1) advanced maternal age: estimated due date at or beyond 35 years of age.
2) high-risk serum screening result: risk of trisomy 21 > 1/270, trisomy 18 > 1/350, or high risk for neural tube defects (AFP >2.5 MoM).
3) increased NT: NT > 3.0 mm at 11–14 weeks of gestation.
4) high-risk NIPT: Z-scores >3 for trisomy 21, 18, or 13.
5) structural abnormalities on ultrasound: including fetal malformations, intrauterine growth restriction (IUGR), facial anomalies, abnormal amniotic fluid volume, single umbilical artery, *etc.*
6) chromosomal abnormality in one parent: including balanced translocations, aneuploidy, mosaicism, and chromosomal polymorphisms.
7) history of pregnancy or childbirth with chromosomal abnormalities: referring to previous pregnancies with aneuploidy, deletions, duplications, *etc.*
8) intellectual disability in one parent: either parent has a history of intellectual disability.
9) monogenic disease: either parent is a carrier or affected by a monogenic disorder.
10) cord blood: The sample was collected from cord blood.
11) adverse pregnancy and birth history: excluding those caused by known genetic factors, including fetal demise, congenital anomalies, spontaneous abortion, biochemical pregnancy, *etc.*
12) others: indications not collected, failed NIPT results, or exposure to teratogenic substances.

Chromosomal results were categorized on four hierarchical levels:

Level 1 – Mosaicism: Abnormal results were classified into mosaic, non-mosaic, polymorphism, and normal. Mosaicism included both numerical and structural chromosomal mosaicisms. Non-mosaic abnormalities included numerical and structural chromosomal abnormalities. Polymorphisms included inv (9), chromosome length polymorphism, satellites, and satellite stalk length variations.
Level 2 – Structural Classification: Chromosomal abnormalities were further classified into: inversion, translocation, duplication, deletion, isochromosome, psu dic, Robertsonian translocation, derived chromosome, additio, structural aberration, aneuploidy, triploid, hermaphroditism, chromosome length polymorphism, and normal. Definitions of inversion, translocation, duplication, deletion, isochromosome, psu dic, Robertsonian translocation, derived chromosome, aneuploidy, and triploid follow the ISCN 2016 guidelines. Karyotype results obtained prior to 2016 were reinterpreted and corrected according to the ISCN 2016 guidelines. Structural aberration refers to aberrations not covered by the other specified categories. Hermaphroditism refers to the co-existence of 46,XX and 46,XY. Chromosome length polymorphisms include heterochromatin variations, satellites, and satellite stalk variations.
Level 3 – Chromosomal involvement: Each abnormality was annotated according to the specific chromosome involved in the structural abnormality or aneuploidy. Hermaphroditism, triploid, and normal karyotypes were annotated as “None”.
Level 4 – Balance status: Abnormalities without loss or gain of chromosomal material were annotated as balanced, while those with deletion or duplication were annotated as unbalanced. In Robertsonian translocations, if there was no loss or gain of long arm, they were marked as balanced. All polymorphisms were considered balanced. Hermaphroditism were classified as unbalanced.

### 2.5 Ethics approval

This study was reviewed and approved in advance by the Ethics Committee of Beijing Obstetrics and Gynecology Hospital, Capital Medical University (approval No. 2017-KY-043-01). All procedures involving human participants adhered to the Declaration of Helsinki 1964 and its subsequent revisions, or other applicable ethical standards. Due to the retrospective nature of the study, the Ethics Committee of Beijing Obstetrics and Gynecology Hospital waived the need of obtaining informed consent.

## 3 Results

### 3.1 Indications for prenatal diagnosis

**FIGURE 2:**
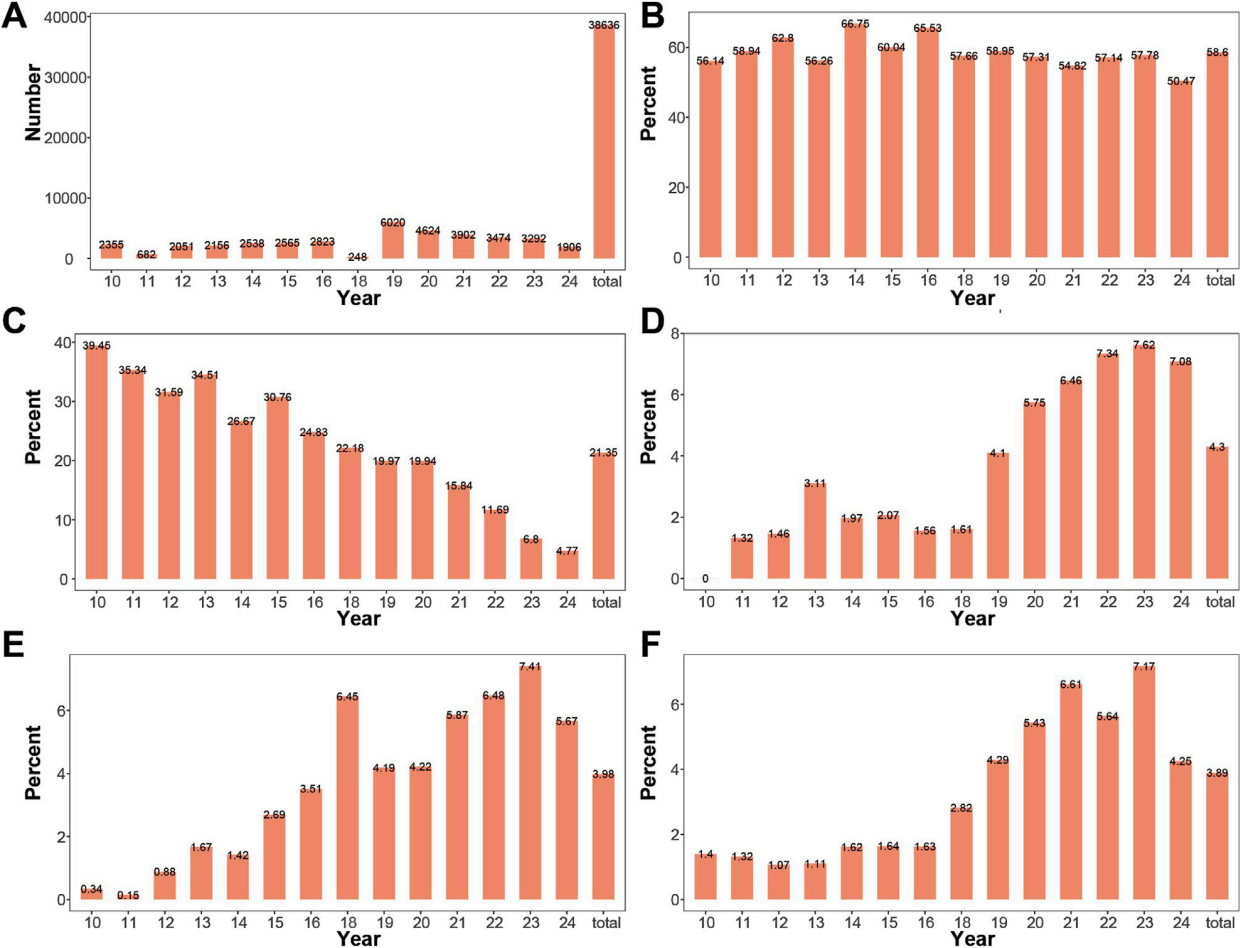
Changes in Amniocentesis Indication Composition by Year **(A)** Number of samples collected from 2010 to 2024. Samples were collected in all years except 2017, with a smaller number collected in 2011 and 2018. **(B)** Proportion of advanced maternal age cases across years. The proportion of advanced maternal age remained relatively stable from 2010 to 2024. **(C)** Proportion of high-risk serum screening result pregnancies across years. The proportion of high-risk serum screening result gradually decreased from 2010 to 2024. **(D)** Proportion of high-risk NIPT cases across years: The proportion of high-risk NIPT gradually increased from 2010 to 2024. **(E)** Proportion of pregnancies with increased NT across years. The proportion of pregnancies with increased NT gradually increased from 2010 to 2024. **(F)** Proportion of pregnancies with ultrasound abnormalities across years. The proportion of pregnancies with ultrasound abnormalities gradually increased from 2010 to 2024.

The study collected a total of 38,652 samples from pregnant women (including failed specimens). The largest number of samples was collected in 2019, with 6,028 cases, followed by 4,627 cases in 2020. The distribution of collected samples by year and the total number of samples is shown in Figure 2A.

Indications for prenatal diagnosis included: advanced maternal age, high-risk serum screening result, increased NT, high-risk NIPT, structural abnormalities on ultrasound, chromosomal abnormality in one parent, history of pregnancy or childbirth with chromosomal abnormalities, intellectual disability in one parent, monogenic disease, cord blood, adverse pregnancy and birth history, and others. Advanced maternal age had the highest proportion, accounting for 58.59% of the total, with the highest proportion in 2014 (66.75%), followed by 2016 (65.53%), and the lowest proportion in 2024 (50.47%). The proportions of high-risk serum screening, high-risk NIPT, increased NT, and abnormal ultrasound findings ranked second, third, and fourth, respectively. The annual and overall changes in the proportions of advanced maternal age, high-risk serum screening, high-risk NIPT, thickened NT, and abnormal ultrasound are shown in Figures 2B–F. The proportion of high-risk serum screening showed a decreasing trend over the years. In contrast, the proportions of high-risk NIPT, thickened NT, and abnormal ultrasound increased over the years.

Chromosomal abnormality in one parent, history of pregnancy or childbirth with chromosomal abnormalities, intellectual disability in one parent, monogenic disease, cord blood, and adverse pregnancy and birth history had relatively low proportions both overall and by year. The number of samples collected each year and the distribution of indications for prenatal diagnosis by year are detailed in Supplementary Table S1.

### 3.2 Non-mosaic cases have the highest proportion across all years

**FIGURE 3:**
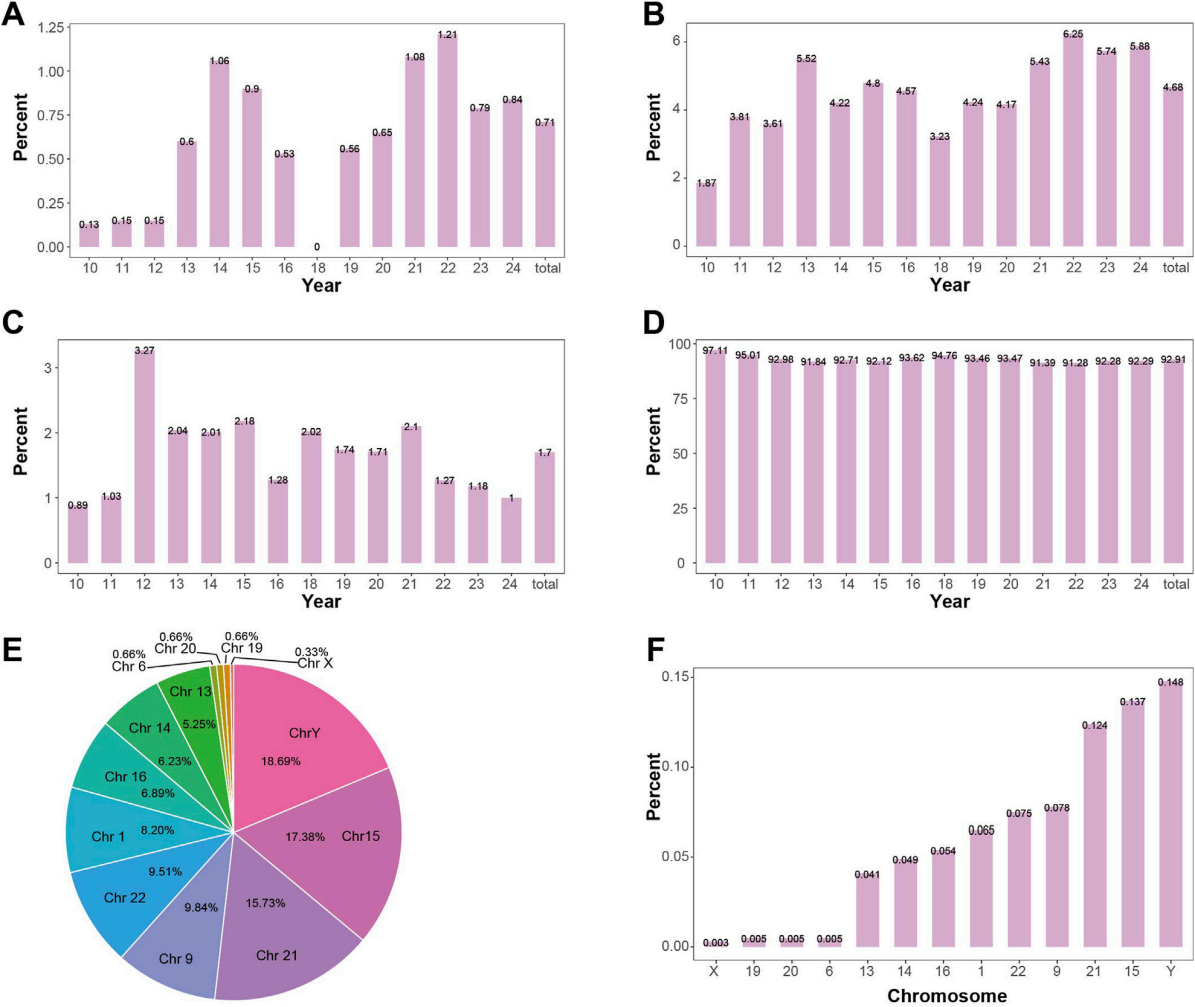
Positive Detection Rates in Mosaic Level **(A)** Detection rate of mosaicism by year. The overall positive rate for mosaicism is 0.71%, showing an increasing trend. **(B)** Detection rate of non-mosaic abnormalities by year. The overall positive rate for non-mosaic abnormalities is 4.68%, showing an increasing trend. **(C)** Detection rate of polymorphisms by year. The overall positive rate for polymorphisms is 1.7%, with the detection rate remaining relatively stable. **(D)** Detection rate of normal samples by year. The overall positive rate for normal samples is 92.91%, showing a decreasing trend. **(E)** The proportion of each chromosome length polymorphisms in the detected length polymorphisms. The Y chromosome has the highest proportion (18.69%), followed by chromosome 15 (17.38%). **(F)** Detection rate of chromosome length polymorphisms in the overall fetus sample. The Y chromosome has the highest detection rate of length polymorphisms (0.148%), followed by chromosome 15 (0.137%).

In this study, a total of 38,652 pregnant women’s samples were collected, with 16 samples failing to produce valid chromosomal karyotype. A statistical analysis was performed on the 38,636 successful samples. The proportions of non-mosaic, mosaic, polymorphic, and normal results in the overall dataset were 4.68%, 0.71%, 1.7%, and 92.91%, respectively. Non-mosaic cases had the highest proportion in 2022 (6.25%) and the lowest in 2010 (1.87%). Mosaic cases had the highest proportion in 2022 (1.21%) and the lowest in 2010 (0.13%). Polymorphic cases had the highest proportion in 2012 (3.27%) and the lowest in 2010 (0.89%). Normal results had the highest proportion in 2010 (97.11%) and the lowest in 2022 (91.28%). The proportions of mosaic, non-mosaic, polymorphic, and normal results across the years are shown in Figures 3A–D. Detailed information on the detection of mosaic, non-mosaic, polymorphic, and normal results is provided in Supplementary Table S2.

### 3.3 Inversion of chromosome 9 is the most common polymorphism

The study analyzed the proportions of different chromosomal abnormalities in non-mosaic, mosaic, and polymorphic cases, as well as the incidence of different abnormalities in the overall population, as shown in Supplementary Table S3.

Among the chromosomal polymorphism types, 353 cases of chromosome 9 were detected, accounting for 53.89% of the polymorphisms, with an incidence rate of 0.91% in the amniocentesis population (353/38,636). Chromosome length polymorphism were detected in 302 cases, accounting for 46.11% of the polymorphisms, with an incidence rate of 0.78% in the amniocentesis population (302/38,636). Among these, the most commonly heterochromatin length polymorphism were detected chromosome Y, followed by chromosome 15. The proportion of length polymorphism in each chromosome and their incidence in the overall population are shown in Figures 3E,F.

### 3.4 Sex chromosome aneuploidy is more likely to exhibit mosaicism

In the study, a total of 1,557 cases of aneuploidy were detected (4.02%, 1,557/38,636), of which 1,528 were simple aneuploidy (3.95%, 1,528/38,636), 25 cases had combined structural abnormalities (0.06%, 25/38,636), 2 cases had combined translocations, 1 case had combined deletion, and 1 case had combined derivative chromosome. Among the 1,557 cases of aneuploidy, 1,338 were detected in a non-mosaic state (3.46%, 1,338/38,636), and 219 were detected in a mosaic state (0.57%, 219/38,636). All aneuploidies with combined abnormalities were found in mosaic cases.

**FIGURE 4:**
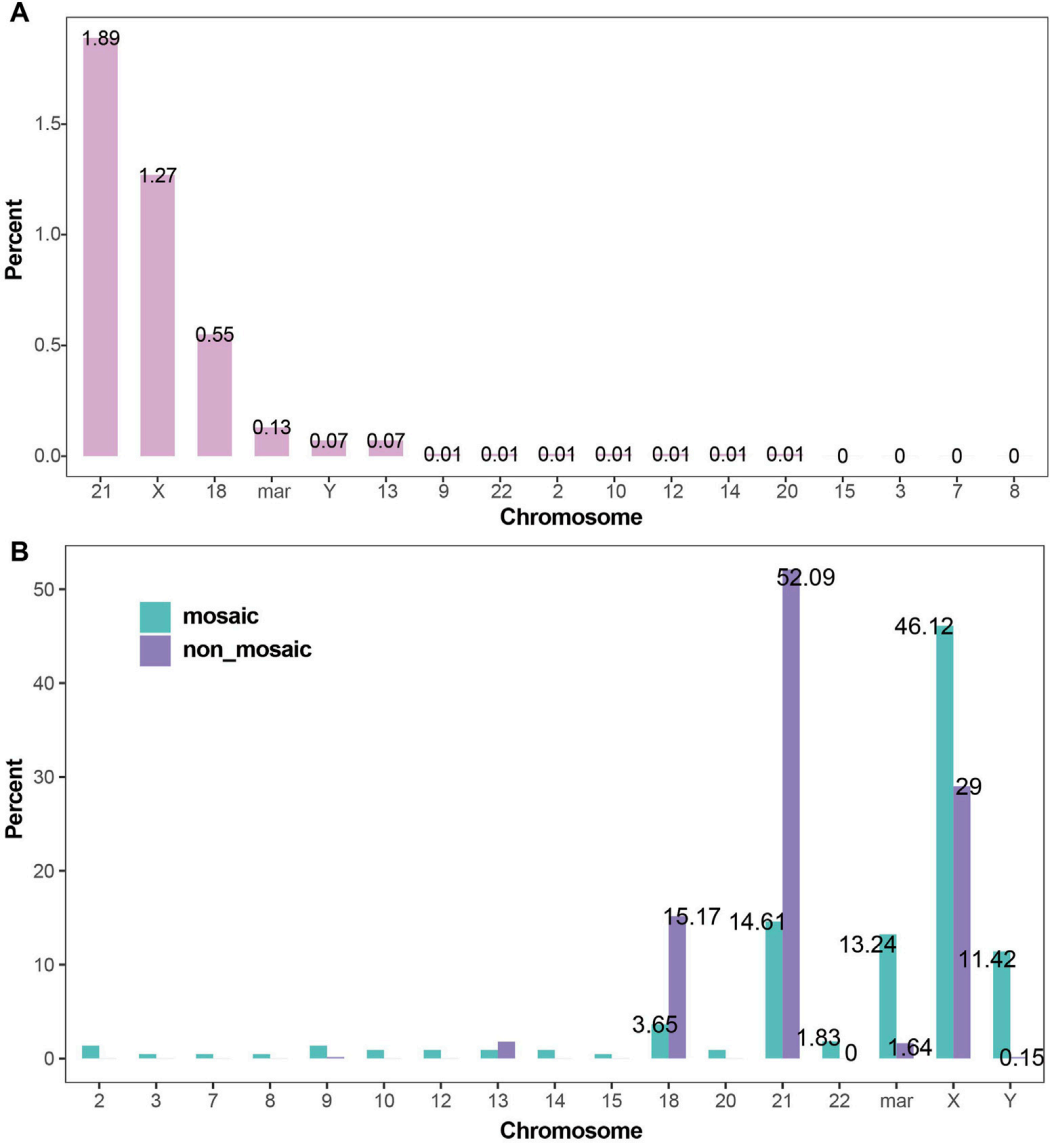
Detection Rates of Chromosomal Aneuploidies **(A)** Detection rates of chromosomal aneuploidies. Trisomy 21, X chromosome aneuploidy, trisomy 18, and marker chromosomes were the most frequently detected aneuploidies, with detection rates of 1.89%, 1.27%, 0.55%, and 0.13%, respectively. **(B)** Proportions of chromosomal aneuploidies in mosaic and non-mosaic cases: Aneuploidies of the X chromosome (46.12% vs. 29.00%), marker chromosomes (13.24% vs. 1.64%), and Y chromosome (11.42% vs. 0.15%) were significantly more prevalent in mosaic cases compared to non-mosaic cases. In contrast, trisomy 21 (52.09% vs. 14.61%) and trisomy 18 (15.17% vs. 3.65%) were more frequently observed in non-mosaic cases than in mosaic ones.

Aneuploidies of chromosomes 21, X, 18, mar, Y, and 13 were the most common. The detection rates of aneuploidy for each chromosome in the amniocentesis population are shown in Figure 4A. The proportion of mosaic and non-mosaic cases for chromosome 21 aneuploidy were 14.61% and 52.09%, respectively. The proportion of mosaic and non-mosaic cases for X chromosome aneuploidy were 46.12% and 29.00%, respectively. The proportions of mosaic and non-mosaic cases for Y, 18, 13, and mar chromosomes are shown in Figure 4B.

### 3.5 Inversion of chromosomes 9 and Y are the most common inversions

**FIGURE 5:**
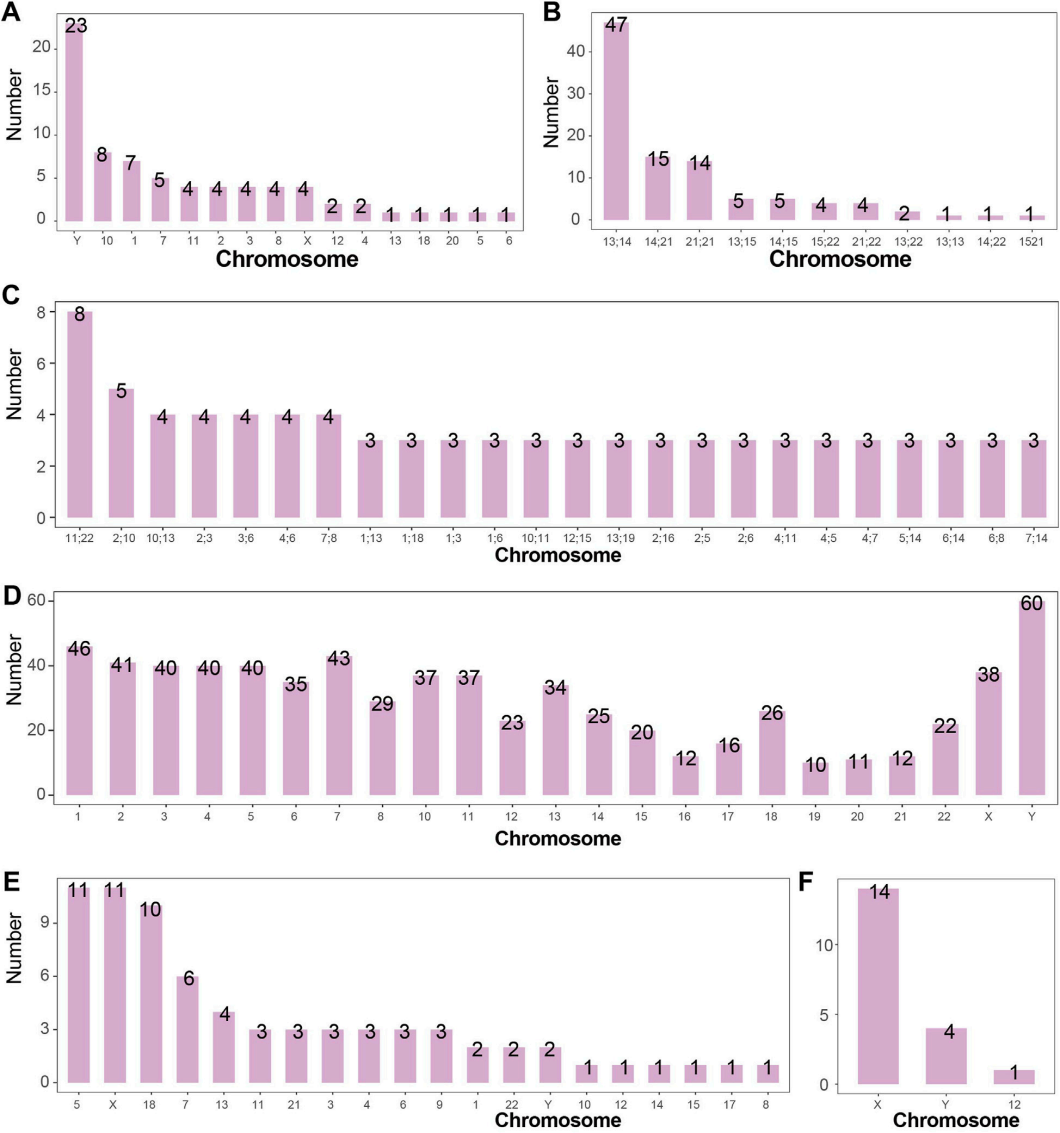
Number of Structural Abnormalities Detected in Each Chromosome **(A)** Number of chromosomal inversions detected. Inversions were most commonly detected in chromosomes Y, 10, and 1, with 23, 8, and 7 cases respectively. Chromosome 9 is not shown. **(B)** Number of Robertsonian translocations detected. The most frequently detected Robertsonian translocations occurred between chromosomes 13 and 14, 14 and 21, and 21 and 21, with 47, 15, and 14 cases respectively. **(C)** Number of chromosomal reciprocal translocations detected. Reciprocal translocations between chromosomes 11 and 22 (8 cases), and between 2 and 10 (5 cases) were the most common. **(D)** Number of chromosomal breaks detected. The Y chromosome had the highest number of breaks detected (60 times). In general, the number of breaks correlated with chromosome size, except for chromosome 18, which exhibited disproportionately more breaks relative to its length. **(E)** Number of chromosomal deletions detected. Deletions were most frequently observed in chromosomes 5, X, and 18, with 11, 11, and 10 cases respectively. **(F)** Number of isochromosomes detected. Isochromosomes were detected in chromosomes X (14 cases), Y (4 cases), and 12 (1 case).

A total of 425 cases of inversions were detected in the study, including 353 cases of chromosome 9 inversion and 72 cases of inversions in other chromosomes. The highest incidence was found for chromosome 9 inversion, followed by inversions on chromosomes Y, 1, 10, and 7, with an incidence of 0.06%, 0.02%, and 0.02%, respectively, in the amniocentesis population. The number of inversions in chromosomes other than chromosome 9 is shown in Figure 5A.

The study also detected 2 cases of chromosome 11 inversion in mosaic states, while all other inversions occurred in non-mosaic states.

### 3.6 Robertsonian translocations are most common between chromosomes 13 and 14

A total of 99 cases of Robertsonian translocations were detected in the study, with an incidence rate of 0.26% (99/38,636) in the amniocentesis population. Among the 99 cases, 2 occurred in mosaic states. The study identified 11 types of Robertsonian translocations, with the most common being between chromosomes 13 and 14 (47/38,636, 0.12%), followed by translocations between chromosomes 14 and 21 (15/38,636, 0.04%). The detection numbers of other types of Robertsonian translocations are shown in Figure 5B.

### 3.7 Reciprocal translocations between chromosomes 11 and 22 are the most common

A total of 218 cases of translocations and derivative chromosomes were detected in the study, with an incidence rate of 0.56% (218/38,636) in the amniocentesis population. Among these, 31 cases occurred in mosaic states and 187 cases in non-mosaic states, with incidence rates of 0.08% (31/38,636) and 0.48% (187/38,636), respectively.

In the 218 cases, 127 types of reciprocal translocations between two chromosomes were identified, with 24 types occurring at least 3 times. The reciprocal translocation between chromosomes 11 and 22 occurred 8 times, with an incidence rate of 0.02% (8/38,636) in the amniocentesis population. The detection numbers of other high-frequency reciprocal translocations between two chromosomes are shown in Figure 5C.

### 3.8 Breakage events are predominantly observed on the Y chromosome and chromosome 1

Breakage events outside centromeric regions were analyzed in this study. A total of 772 fetuses were identified with chromosomal breakage events, among which 425 cases involved inversions. Of the 772 breakage events, 353 were polymorphic (46,XN,inv (9)), 359 occurred in non-mosaic cases, and 60 were observed in mosaic cases. In total, 1,434 breakage events were detected. Chromosome 9 was involved in 737 breakage events, making it the most frequently affected chromosome. Excluding polymorphisms, the Y chromosome and chromosome 1 showed 60 and 46 breakage events respectively, ranking as the most commonly involved chromosomes. Breakage events involving chromosomes other than chromosome 9 are summarized in Figure 5D.

### 3.9 Deletions most frequently occur on the X chromosome and chromosome 5

A total of 72 deletions were identified in this study, with an overall incidence of 0.19% (72/38,636) among fetuses undergoing amniocentesis. Among these, 8 cases occurred in mosaic fetuses (8/38,636, 0.02%). Deletions were most frequently detected on the X chromosome (11 cases, 0.03%), chromosome 5 (11 cases, 0.03%), and chromosome 18 (10 cases, 0.03%). Deletions involving other chromosomes are summarized in Figure 5E.

### 3.10 Isochromosomes primarily occur on the X chromosome in mosaic form

A total of 19 isochromosomes were identified in this study, with an overall incidence of 0.05% (19/38,636) among fetuses undergoing amniocentesis. Of these, 6 occurred in non-mosaic cases and 13 in mosaic cases. The X chromosome was involved in 14 cases (0.04%, 14/38,636). Isochromosomes involving other chromosomes are summarized in Figure 5F.

### 3.11 The highest positive rate for unbalanced chromosomal abnormalities is observed in high-risk NIPT groups

A total of 1,742 cases of unbalanced chromosomal abnormalities were detected in this study, with an overall incidence rate of 4.51% (1,742/38,636) among fetuses undergoing amniocentesis. The highest positive rate of unbalanced abnormalities was observed in the high-risk NIPT group (41.49%, 690/1,663), followed by the cord blood (20%, 1/5), intellectual disability in one parent (12.5%, 1/8), and the increased NT group (7.68%, 118/1,536). The detection rates of unbalanced translocations across different clinical indications are detailed in Supplementary Table S4.

### 3.12 Network query of chromosomal abnormalities

**FIGURE 6:**
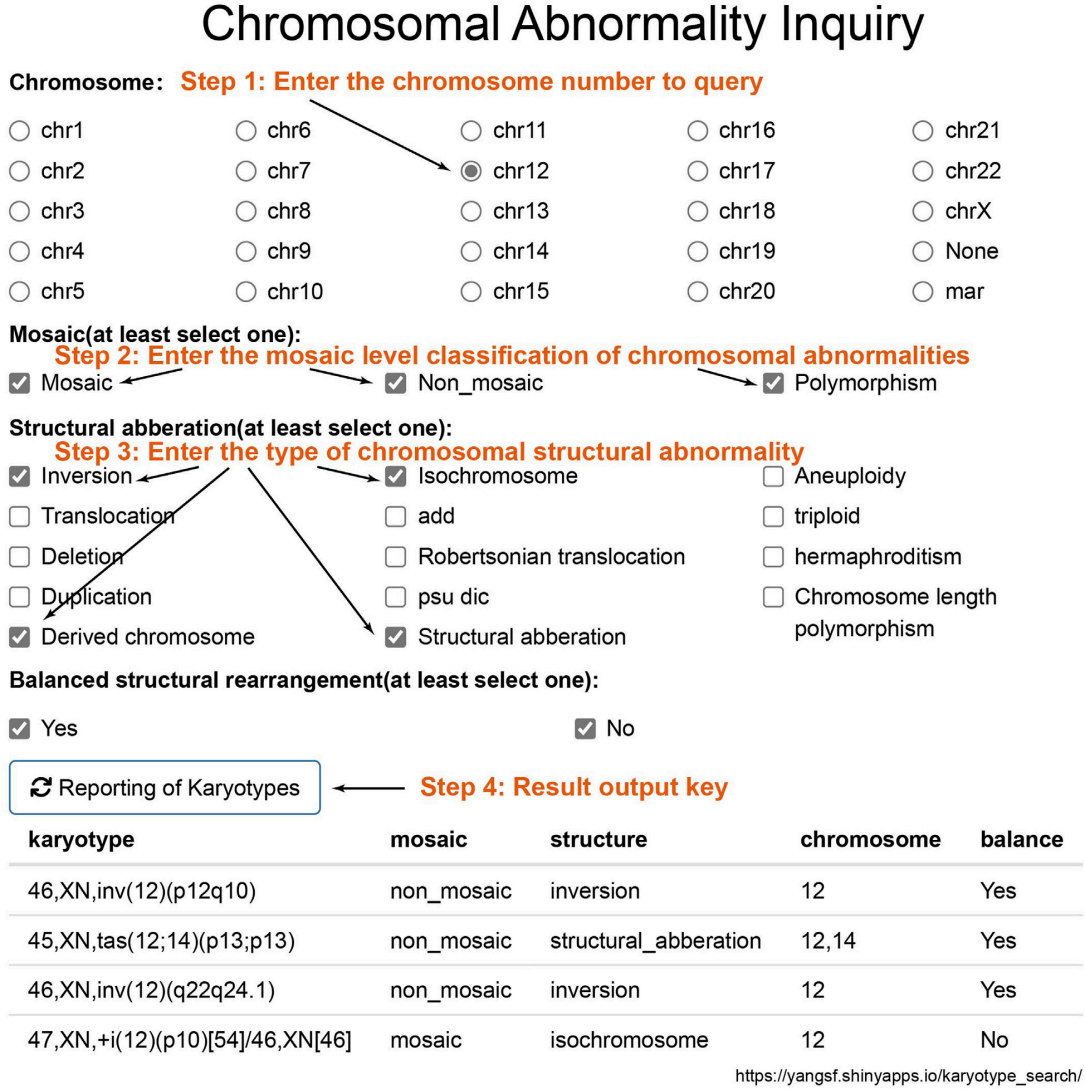
Chromosomal Result Query Interface. To query chromosomal results, sequentially input the chromosome number, mosaicism classification, structural abnormality classification, and whether the abnormality is balanced. Click on “Reporting of Karyotypes” to obtain the chromosomal abnormal karyotype results detected in this study.

In this study, the identified chromosomal karyotype results can be queried through the website (https://yangsf.shinyapps.io/karyotype_search/). As shown in Figure 6, during the query process, users can sequentially input the chromosome number, mosaicism level, chromosomal structural abnormality level, and whether the abnormality is balanced. By clicking “Reporting of Karyotypes,” the chromosomal abnormality karyotype results detected in this study will be displayed.

## 4 Discussion

The detection rate of chromosomal abnormalities in amniotic fluid karyotypes is strongly influenced by the indications for prenatal diagnosis. Different indications warrant distinct genetic testing strategies. For example, whole‐genome sequencing and whole‐exome sequencing yield higher diagnostic rates in cases with ultrasound‐detected structural anomalies (Qi et al., 2024). Indications for prenatal diagnosis vary significantly across studies. In the study by Yanmei et al., high-risk serological screening (36.7%) and advanced maternal age (33.7%) were the most common indications (Zheng et al., 2024). Similarly, Miyuki et al. reported advanced maternal age (54.7%) and high-risk serological screening (18.45%) as the predominant indications (Nishiyama et al., 2015). In our study, we retrospectively analyzed 38,652 prenatal diagnostic cases collected over a 15-year period in Beijing. Through comprehensive statistical analysis, we characterized the evolving spectrum of amniocentesis indications in our region, thereby providing an empirical foundation for optimizing genetic testing strategies. Taken together with previous studies, our findings suggest that indications for prenatal diagnosis are influenced by temporal trends, geographic regions, and technological advancements. For instance, the widespread clinical adoption of non-invasive prenatal testing (NIPT), alongside increasing maternal age, has significantly reshaped the distribution of amniocentesis indications over time.

Mosaicism, defined as the coexistence of two or more genetically distinct cell lines within a single individual, was observed in 0.71% of cases in this study, consistent with previously reported rates (0.49%–1.71%) (Xu et al., 2025; Zhang et al., 2021; Younesi et al., 2021). We further discovered a pronounced chromosomal bias in the occurrence of mosaic *versus* non-mosaic aneuploidies. Chuang et al. similarly reported in preimplantation genetic diagnosis that aneuploidies predominate on shorter chromosomes and mosaics on longer ones (Chuang et al., 2020). Numerous adult studies have linked mosaic loss of chromosome X and Y to increased risks of atrial fibrillation, premature mortality, and various cancers (Lim et al., 2024; Fukami and Miyado, 2022; Zeiher and Braun, 2022; Sano and Walsh, 2024). Our findings indicate that such mosaic losses can originate prenatally. Given their stem‐cell-like properties, amniotic fluid–derived cells may serve as an invaluable *in vitro* platform for investigating mosaic loss of sex chromosomes.

Our study also analyzed the incidence of chromosomal aneuploidies. Consistent with previous reports, trisomy 21 (0.8%–1.89%), trisomy 18 (0.23%–0.55%), trisomy 13 (0.04%–0.13%), and sex chromosome aneuploidies (0.30%–1.34%)—though influenced by indications for prenatal diagnosis—remain the most commonly detected chromosomal abnormalities in prenatal settings (Nishiyama et al., 2015; Younesi et al., 2021; Mademont-Soler et al., 2011; Zhu et al., 2016; Xiao et al., 2016; Evans et al., 1999). Previous studies have provided limited insight into the relationship between structural abnormalities and specific chromosomes. Therefore, we systematically analyzed the chromosomal distributions of structural aberrations, thereby addressing gaps in earlier amniotic fluid karyotype research.

We stratified amniotic fluid karyotype results into four hierarchical levels to dissect the occurrence patterns of mosaicism and structural abnormalities in prenatal diagnosis. Nevertheless, our study has several limitations: (1) Incomplete data capture for certain years. (2) Grouping polymorphisms alongside mosaic and non-mosaic abnormalities for analytical convenience, which may blur distinctions. (3) Summarizing breakpoint locations solely by chromosome number, potentially obscuring more granular locus‐specific trends. To mitigate the latter, we have compiled all aberrant karyotypes identified in our cohort into a dedicated, searchable database with an intuitive retrieval interface (Figure 6) to aid laboratory personnel. (4) CMA or CNV-seq can improve the diagnostic accuracy of mosaicism and detect submicroscopic deletions and duplications (Wang J. et al., 2022; Zhuang et al., 2024; Hao et al., 2020; Xu et al., 2025; Zhang et al., 2021). The integration of conventional karyotyping with molecular cytogenetic methods will greatly enhance future clinical practice and research. However, this study lacks a comparative analysis between karyotyping results and those obtained from CMA or CNV-seq.

In summary, by examining 15 years of amniocentesis indications and karyotype outcomes at our center, we have demonstrated that specific chromosomes are predisposed to particular types of anomalies. Our study elucidates the relationships among inversions, polymorphisms, breaks, deletions, isochromosomes, mosaicism, and their chromosomal contexts. Our findings fill the gaps in previous studies of amniotic fluid karyotype results. Additionally, the searchable database we provide will facilitate rapid reference to chromosomal abnormalities. These insights underscore the importance of targeted attention to certain chromosomes during karyotype analysis in prenatal diagnosis.

## Data Availability

The original contributions presented in the study are included in the article/Supplementary Material, further inquiries can be directed to the corresponding authors.
